# Collective patient behaviours derailing ART roll-out in KwaZulu-Natal: perspectives of health care providers

**DOI:** 10.1186/1742-6405-10-20

**Published:** 2013-07-19

**Authors:** Janet Michel, Christina Matlakala, Rene English, Richard Lessells, Marie-Louise Newell

**Affiliations:** 1 Africa Centre for Health and Population Studies, University of KwaZulu-Natal, Somkhele, PO Box 198, Mtubatuba, KwaZulu-Natal, 3935, South Africa; 2 Faculty of Medicine, University of Southampton, Southampton, UK; 3 University of South Africa, Preller St., Pretoria, 0002, South Africa; 4 Health Systems Trust, Westville, South Africa

**Keywords:** Patient behaviour, ART roll-out, Health care provider perspective, Non-governmental organisation, KwaZulu-Natal

## Abstract

**Background:**

Antiretroviral therapy (ART) roll-out is fraught with challenges, many with serious repercussions. We explored and described patient behaviour-related challenges from the perspective of health care providers from non-governmental organisations involved in ART programmes in KwaZulu-Natal, South Africa.

**Methods:**

A descriptive case study design using qualitative approach was applied during this study. Data was collected from nine key informants from the three biggest NGOs involved in ART roll-out using in-depth semi-structured interviews. Transcribing and coding for emergent themes was done by two independent reviewers. Ethical approval for the study was granted by the UNISA research ethics committee of The Faculty of Health Sciences. Written consent was obtained from directors of the three NGOs involved and individual audio taped informed consent was obtained from all study participants prior to data collection.

**Results:**

Findings revealed six broad areas of patient behaviour challenges. These were patient behaviour related to socio-economic situation of patient (skipping of medication due to lack of food, or due to lack of transport fees), belief systems (traditional and religious), stigma (non- disclosure), sexual practices (non-acceptability of condoms, teenage pregnancies), escapism (drug and alcohol abuse) and opportunism (skipping medication in order to access disability grant, teenage pregnancies in order to access child grant).

**Conclusion:**

New programmes need to address patient behaviour as a complex phenomenon requiring a multi-pronged approach that also addresses social norms and institutions. In the face of continued ART scale up, this is further evidence for the need for multi-sectoral collaboration to ensure successful and sustainable ART roll-out.

## Background

The HIV epidemic has had a major impact on the lives of people in Africa and understanding behavioural responses to HIV is crucial for predicting the future path of the epidemic, for preventing its future spread and maximizing efficient use of limited resources [1-5]. Human behaviour refers to a range of actions exhibited by humans and influenced among other things by culture, attitudes, emotions, values, ethics, authority, rapport, hypnosis, persuasion, coercion and genetics [6]. In the absence of an effective government response in South Africa, many NGOs were established solely to address HIV treatment and care. In KwaZulu-Natal (KZN) province, three major non-governmental organisations (NGOs) led the roll-out of HIV antiretroviral treatment (ART), some independently and others in partnership with the Department of Health [7]. These NGO’s are thus of interest for exploring the challenges experienced in the delivery of ART. ART aims to reduce morbidity and mortality and improve quality of life, through durable suppression of viral replication and restoration of immunologic function, [8,9]. Anything that hinders any of the above processes, and in particular interferes with adherence to the therapy, poses challenges to ART roll-out. Successful administration of ART requires the patient to adhere for life for at least 95% of the treatment prescribed. ART adherence has been found to be influenced by health service- and patient-related factors [10,11] and multiple patient behaviours can collectively enhance risks of HIV infection acquisition, transmission and treatment non-adherence [12,13]. Suboptimal adherence leads to incomplete viral suppression, continued destruction of the immune system, disease progression, emergence of resistant viral strains, and thus limits future treatment options [3,8,14].

In South Africa, ART scale up started in 2004 and in 2010 the South African Department of Health embarked on a large scale ART roll-out programme providing HIV treatment and care to all citizens who need it free of charge [15], with need defined by specific eligibility criteria. Because patient behaviour and adherence is crucial to the success of ART roll-out, we explored and described patient behaviour-related challenges reported from the perspective of non-governmental organisations in KwaZulu-Natal. Much has been written on sexual behaviour, especially sexual disinhibition and the inconsistent use, or unacceptability, of condoms derailing the success of ART [16-18] and there has been mention, but no collective summary, of patient behaviour-related challenges derailing ART from the health provider perspective and this study attempts to address this gap in the knowledge base.

## Results

Participants revealed different patient behaviours they considered detrimental to ART roll-out. Six broad areas emerged as patient behaviour related to socio-economic situation, belief systems, stigma, sexual practices, escapism and opportunism. A summary list of identified themes is given in Table 1.

**Table 1:** Summary of findings using classification by theme and category

**Theme**	**Category**	**Meaning unit**
**Socio-economic situation of patient**	**Poverty**	• Lack of food
		• Lack of transport fees
**Belief system**	**Religious ideological/traditional**	• Self-ordained religious prophets
		• Use of traditional medicine.
**Stigma**	**Non-disclosure**	• Non-disclosure by women
		• Non-disclosure of parents to their teenage children who are HIV positive.
		• Non-disclosure of mothers when they leave the children in the care of grandmothers/ baby minders
**Sexual practice**	**Culture/psychosocial factors**	• Unacceptability of condom use.
		• Teenage pregnancies
**Escapism**	**Substance abuse**	• Alcohol abuse
		• Drug abuse (whoonga)
**Opportunism**	**Abuse**	• SASSA guidelines.
		• Child grant

### Socio-economic situation related behaviour

Socio-economic problems of the patients were indicated as presenting a real challenge to the successful roll-out of ART. Factors related to the socio-economic situation of the patients were identified as aspects such as skipping of medication doses when food is unavailable and inability to collect medication due to lack of transport fees. The following were some of the narratives from the participants in relation to skipping medication:

*“Socio-economic problems lead patients to skip doses of medication when food is unavailable”.* (Professional nurse 1)

*“Social problems lead to defaulting treatment. Many patients lack support at home. They miss review dates due to lack of transport fee and they do not take medicine when they do not have food. They say the tablets make one very sick when taken on an empty stomach”. *(Professional nurse 3)

The findings concur with research done elsewhere [19] that revealed food insecurity as a common and important barrier to accessing medical care and ARV adherence.

*“Patients discontinue treatment when there is lack of money for transport to collect medication supplies”.* (Programme coordinator 1)

The findings are also in line with Du Preez [20] who indicated that some of the problems hindering the fight against HIV/AIDS are unemployment and poverty; and that despite HIV treatment being free, transport costs are a major obstacle for people on ART.

### Belief systems

All participants mentioned how patients’ belief systems are interfering with ART use. A belief system can be religious, philosophical, ideological or a combination of these. It is a way of life, basis of culture, identity and moral values [21]. All the participants mentioned use of traditional medicine and religious prophets as systems people frequently fall back on to regain wellness and equilibrium. Cultural traditions help participants cope with illness, physical disability, economic hardship, and face oppression and discrimination [22]. The participants revealed the following:

*“60-70% of the patients mix traditional herbal medicine and ART”*. (Professional nurse 1)

“*Enemas given to children impair the absorption of ART”*. (ART Doctor 2)

*“We had cases of inpatients that died clearly from muti* [African traditional medicine] *over-dose. One had this blackish thing that got stuck in his throat and was pulled out during resuscitation”*. (ART Doctor 3)

Use of traditional medicine is not the only belief system the participants identified as posing a challenge to ART roll-out as revealed by the following further quotes:

*“A patient disappeared to visit one of the prophets in Mpumalanga and later came back with a very low CD4 cell count and died a few days later”.* (Professional nurse 2)

*“One patient was doing very well on treatment. She asked for leave to go home and was granted. Her CD4 cell count was 520 when she left. She returned some weeks later after having spent most of that time at a prophet”s shrine in Johannesburg with a CD4 cell count of 28. We tried all we could but she died within a week”.* (Professional nurse 3)

### Stigma

Non-disclosure for fear of stigma was highlighted as a challenge by most of the participants. One professional nurse explained the fear of stigma as follows:

*“Patients refuse to be referred to local clinic. We have staff members working here in an organisation that rolls out ART but people are unwilling to go present themselves at the clinic to get ART. Someone said I would rather die than to go to any of these clinics because the nurses know me”.* (Programme coordinator 1)

*“Most patients do not want to be referred to local clinic, something that could help considering their financial problems. They would rather travel 50 km every month to take supplies than be seen at their local clinic”.* (ART Doctor 3)

The above statement indicates that health care providers perceive stigma as still deep despite public sector campaigns [23]. Health care workers working in ART programmes seem themselves not to have gotten over stigma let alone patients seeking care from these providers as revealed above. The participants mentioned that stigma manifested in the form of non-disclosure of different groups like women not disclosing to men. One ART doctor made the following comments:

*“Most women who are dependent on their boyfriends do not disclose for fear of loss of income”.* (ART Doctor 3)

Non-disclosure of mothers to baby-minders like grandmothers was cited by most participants. One professional nurse described this as follows:

*“Baby dumping at grandmothers who are oblivious to the baby’s HIV status especially in teenage pregnancies is a problem. If they did not know the status of the baby, how on earth do you expect them to have understood the importance [of ART] and adhered to the treatment as required?”* (Professional nurse 2)

Non-disclosure of parents to their (teenage) children who are HIV positive was mentioned by most participants as a real concern exacerbated by the very fact that puberty itself is a phase fraught with its own challenges.

*“Parents do not disclose to their HIV positive teenagers making it difficult to explain to this group what medication they are taking”.* (Professional nurse 1)

### Sexual behaviour

Sexual behaviour is defined as the manner in which humans experience and express their sexuality. People engage in a variety of sexual acts from time to time, and for a wide variety of reasons. Teenage pregnancies and unacceptability of condoms were cited by most participants. One of the ways of preventing HIV is through the use of condoms. The participants cited this as a big challenge especially in light of the amount of money that has been poured into making condoms freely available. Condom use seemed not to have been embraced in KwaZulu-Natal. One Doctor commented:

*“Use of condoms has not been widely accepted in this community. They come back with STIs and yet claim to use condoms”.* (ART Doctor 1)

Teenage pregnancies were cited as a huge challenge by most participants:

*“70-80% of the pregnant women are teens. The children do not see the importance of education”*. (Professional nurse 1)

*“Desperation amongst teenage mothers is overwhelming. Crisis centre in Pinetown is full and cannot accommodate more desperate teenagers”*. (Programme coordinator 1)

The problem of teenage pregnancies pose additional challenges to ART roll out as put in by one participant:

*“Post-delivery, girls go back to school. They seldom come back for review, losing both mother and baby as loss to follow up”.* (Professional nurse 3)

The health workers highlighted that the interactions between schooling, sexual debut, pregnancy, child grant and social desirability of having a baby, especially since it is common for girls to continue school after the birth of a child, is not understood adding to the complexity.

### Escapism

Most participants mentioned that some patients try to escape reality through substance abuse. Escapism is defined as the tendency to seek distraction and relief from unpleasant realities, especially by seeking entertainment or engaging in fantasy [24]. This could be a sign of failing to cope with their situation or status. Alcoholism was cited as one of the escape routes patients were using as revealed below:

*“A number of males patients often abuse alcohol. They say it helps them forget about their problems”.* (ART Doctor 2)

*“We have some patients who come to pick up their supplies totally drunk. One wonders how they can adhere to such a strict treatment”*. (Programme coordinator 2)

Drug abuse, particularly ‘whoonga’, was cited by all the participants as a new challenge that has crept into the ART arena. ‘Whoonga’ is a highly addictive substance incorporating efavirenz, a commonly used antiretroviral drug usually mixed with marijuana or other illicit drugs [25]. In KwaZulu-Natal, drug dealers are now enticing AIDS patients into selling their life-saving antiretrovirals [26]. Drug abuse was cited as the other escape route as revealed by the following statements from participants:

*“Many patients come back to claim more drugs after allegedly having been robbed. In some settings spouses share one month’s supply and sell the other supply to drug dealers. They then come back with an affidavit from the police and demand another drug supply”.* (Programme coordinator 3)

*“Some patients have been mugged. They were followed from the hospital and when they alighted from the taxi, they were told to hand over their hand bags for something important was in there. All the muggers took out were ARVs”*. (Professional nurse 1)

*“They stand at the gate and scout for candidates that are likely coming for ART supply. The thin and sickly are targeted and followed”.* (ART Doctor 2)

### Opportunism

All the participants mentioned that South African Social Security Agency (SASSA) guidelines were fuelling poor adherence as the patients were eager to access the grant. SASSA guidelines stipulate the criteria to be used for an HIV positive patient to receive a grant. One criterion used previously was a CD4 cell count of 200 and below (it should be noted that the criterion has now changed as explained below). An opportunist is defined as a person who adapts his actions to take advantage of the circumstances with little regard for consequences for others [27]. The study revealed that in order to access a perceived benefit, patients do not always act in their own best interests [28]. Participants also revealed what has been shown in research elsewhere that most patients say: “It is better to die from AIDS than to die of hunger [29].

The following statements came from participants:

*“Patients skip their medication in order to keep the CD4 count low thereby making them eligible for the SASSA grant. SASSA guidelines are fuelling non adherence”.* (Professional nurse 2)

*“Patients threaten us with unspecified consequences if we do not recommend them to receive a grant. Alternatively, they simply stop taking medication until the CD4 cell count has dropped below 200”.* (Professional nurse 3)

The grant eligibility criteria have since changed to the clinical picture of the patient irrespective of CD4 count. The discretion is now in the hands of the doctor and they still report pressure from patients to recommend them for a grant.

Teenage pregnancies were also cited as an opportunistic practice, demonstrating the complex nature of patient behaviour in ART as well as making it difficult to define where opportunistic behaviour or any another patient behaviour like sexual begins or ends. It was further speculated that the child grant could be peddling this trend of teenage pregnancies. Some participants mentioned the following about teenage pregnancies:

*“There are fifteen year old girls coming for the third pregnancy. They say with three kids you can get a decent amount of support grant to live on”. *(Professional nurse 1)

**Figure 1:**
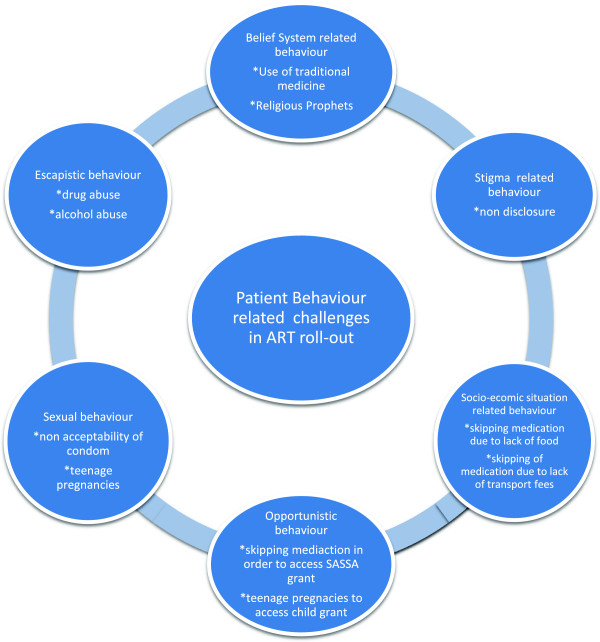
Diagrammatic summary of findings.

It is worth mentioning that Makiwane and Udjo (2006) [30] found no link between teenage fertility and child support grant. More research is called for into this area since little is known about what is enticing teenagers to fall pregnant despite knowledge and free availability of family planning methods (Figure 1).

## Discussion

Studies on patient behaviour to date have mostly dwelt on sexual behaviour in ART roll-out [31-35]. This study revealed that patient-related behaviours undermining ART roll-out are not only confined to sexual practices like unacceptability of condoms, but go beyond that and that these behaviours are heavily influenced by broader socioeconomic, cultural, and environmental factors [12].

Findings indicated that the socio-economic situation of patients has a bearing on the success or otherwise of ART programmes. Patients skip medication doses when they do not have food because ARVs increase appetite and lead to hunger in the absence of food, the side effects of ARVs are exacerbated in the absence of food and doses are skipped or ARVs not started at all if there is added nutritional burden. In addition patients do not pick up their monthly medical supplies if they do not have transport fees. Taking the socio-economic aspects of the population or community into consideration before rolling out any programme is of utmost importance to ensure efficiency and effectiveness. Measures to ensure food security and strategies like mobile ART teams that bring the service into the community should be taken into consideration before the actual roll-out of ART begins.

Findings indicated that use of traditional medicine and religious prophets are systems people frequently fall back on to regain wellness and equilibrium. The results support previous research that revealed that cultural traditions help participants cope with illness, physical disability, economic hardship, and face oppression and discrimination [22,36]. According to the World Health Organisation, 80% of Africa’s population uses traditional medicine for primary health care [37]. Considering the proportion of the native population that seeks help from traditional healers, this challenge is of utmost importance in ART roll-out. Van Dyk (2001) [38] mentions the need to take into consideration ancestors, witches, sorcerers, importance of having children, perception of condoms, the importance of community life and traditional healers when planning HIV and AIDS programmes keeping in mind that these patients may not fully disclose other therapies[39] . Of concern to the health care providers are toxicities as has been demonstrated in other studies [36,40]. Findings revealed that belief systems, religious and ideological being rooted in and intertwined with culture, pose challenges to health care workers as health education on the dangers of mixing ART and traditional medicine goes unheeded and self-ordained religious prophets tell patients that they have been prayed for and are now healed and need not take medication putting themselves, close contacts and family at risk especially with MDR TB [41]. The health care workers could not ascertain whether patients take traditional medicine as a result of their socio- economic situation (financial and transport barrier) or do they do so because traditional and complementary medicine is so embedded in culture. In light of this we recommend the integration of traditional healers and faith institutions into the health system as partners in HIV treatment and care. The South African government has recognized this need with the Draft National Policy on African traditional medicine in 2008 but the integration of TM/CAM into public and private facilities and the reimbursement by medical schemes is far behind [42].

Stigma was also cited as a challenge, concurring with previous research that revealed that HIV related stigma and discrimination remain a key concern in South Africa despite the multitude of HIV awareness campaigns by government and civil society organizations [23,43]. The intimate nature in which HIV is transmitted has contributed to the extreme levels of stigma and discrimination surrounding those infected by the virus [35].) The situation is compounded if children are involved [43]. The economic dependency of women on men and threat or fear or rejection could be to blame [44,45]. This is further evidence to show that if the women do not disclose their status, protected sex or condom use is most likely not going to be considered, increasing the chances of HIV transmission. This underscores the importance of promoting and improving the status of women [46] in the community through education, skills training and creation of employment opportunities. Non-disclosure of parents to their (teenage) children who are HIV positive was revealed to be of concern to the health care providers as this group is in the stage of exploring sexual relations, bringing to the fore the need to address the whole issue of ‘positive prevention [47,48].

Findings indicated that the health care providers perceive condom acceptability to be low in the community. This is in line with other studies that revealed that despite millions of Rand having been poured into promoting the use of condoms to prevent the spread of HIV, condom use is still relatively low [16-18]. The current approach to reduce HIV transmission through condom use appears to have borne little fruit [16,49]. What makes it complex is that in other studies, food insecurity was found to decrease control over condom use and increased high risk sexual behaviour among HIV-infected individuals [19,50] demonstrating complex links between sexual behaviour and socio-economic and cultural environments. Could this be further evidence to the premise that behaviour is so rooted in social contexts, so inflected by social differences and so at the mercy of social resources that behaviours must be thought of as primarily social subject to individual variations at the margins only? Is this a suggestion that there are therefore more insidious and powerful determinants of behaviour that need addressing [12,51]? It is important that organisations in ART roll-out take into consideration the fact that condom availability does not necessarily translate to condom acceptance let alone use. This calls for a combination of strategies in HIV prevention including programs targeting individual behaviour; broad-based efforts to alter social norms and address the underlying drivers of the epidemic; and effective use of biomedical or technological tools, such as treatment of sexually transmitted infections (STIs), medical male circumcision and Treatment as Prevention [52]. Combination prevention is essential since HIV prevention is neither simple nor simplistic [12].

Results suggested that some providers think that patients try to escape reality through substance abuse, a sign of failing to cope with their situation or status. Health care providers think that these patients see their situation to be so beyond what they can deal with so much so that they see an exit from this state as the only way to bring relief hence they resort to alcohol and drugs. Persons living with HIV have high rates of co-morbid depression, estimated to be between 20-50%, and according to literature, depression predisposes to risky behaviour [39,53]. The findings suggest that it is most likely during this period of distorted perception of risk that HIV is transmitted, re-infections occur and medication get forgotten. This is further evidence to the importance of screening HIV infected people for depression and mental health. Department of Health and organisations involved in ART roll-out need to be aware of the importance of routine screening patients for depression [53-55] and the detrimental vicious cycle this can cause in ART, a gap the current ART tools have not filled. While the health care workers invest in teaching the importance of protected sex, a depressed patient on the other hand due to his illness, might not even see the risk the health care worker is talking about as a result of the impaired risk perception [35,56]. We conclude that it is this sense of hopelessness that makes them not only prone to risky behaviour but also makes them not to adhere to treatment for nothing seems to matter anymore, be it their life or some-one else’s life. The critical staff shortage in the health system [35,36] and the fact that most clinics in South Africa do not provide mental health services, compound this challenge for health providers. Many patients according to the health care providers are dying for care, love and hope. Unfortunately they search in the wrong places. Heavy alcohol use and stimulants remain major drivers of HIV transmission in many places and in many groups of people [12]. Drug and alcohol abuse are factors that threaten adherence in ART [57].

Over and above the issues of adherence, health care providers are concerned by the fact that alcohol increases susceptibility to some infections. Infections associated with both alcohol and AIDS include tuberculosis, pneumonia, hepatitis C, which is a leading cause of death among people living with HIV. Alcohol may also increase the severity of AIDS -related brain damage which is characterised in its severest form by profound dementia and a high death rate [58,59]. The patients drink to forget their problems but they tend to forget their medication too, a major challenge in ART where at least 95% of adherence is required for treatment to be successful. Religiosity may foster values, beliefs, and norms that reduce avoidant coping behaviours such as drinking denial and withdrawal [60]. Studies have shown that more religious individuals are less likely to engage in negative health behaviours such as drinking and smoking [61,62]. Faith organisations could play a critical role in ART roll-out by providing hope, re -emphasizing the value and sanctity of every life and most of all providing love and care the health care workers cannot provide due to staff shortages [60].

The problem of drug abuse (whoonga) was revealed as being rife in and around Pinetown, near Durban. This problem was also expressed in the media and a documentary film was recorded by local television [25] and radio [63] which reported that more and more children as young as 11 are smoking whoonga no longer to get high, but to avoid the pain experienced when the drug levels drop. High HIV prevalence rates have resulted in large numbers of children growing without parents [64,65]. Lack of parental support, monitoring and communication, psychosocial factors like family environment, a common feature in communities ravaged by HIV and AIDS could be contributing [46,66]. In light of this, the involvement of community, politicians, leaders, business people and social institutions is called for to deal with this challenge comprehensively and synergistically in a way that is suitable and sustainable in the South African context.

The study also revealed that in order to access a perceived benefit, patients do not always act in their own best interests [28]. SASSA guidelines stipulate the criteria to be used for an HIV positive patient to receive a grant. One of the criteria previously was a CD4 cell count of 200 and below or recently serious clinical disease. It seems to defy the logic of the health care providers that patients forego their health in-order to access a monetary good (grant). This concurs with findings elsewhere that revealed that TB-ridden residents in Khayelitsha charge R50 to R100 for sputum samples to desperate unemployed people to dupe doctors into getting them onto the social grant system [67]. The struggle seems to focus on surviving the day and hopefully see tomorrow. Many participants reported what has been found elsewhere in research that patients say “It is better to die from AIDS than to die of hunger [29]. Talking about a disease that can kill one after 5 or so years seems not taken heed of when the patient is concerned about whether or not he/she can have a meal today. The results are consistent with recent findings that found that unmet subsistence needs are stronger predictors of poor health and adherence to ART [68]. Telling them to preserve a life one does not have becomes a useless exercise. Once again the health care workers found it difficult to define where socio-economic, sexual or opportunistic patient behaviour begins or ends. These behaviours seem interconnected at many levels. Providing free ART does not mean treatment adherence. Multi-pronged approach to deal with the socio- economic context of the patients to meet subsistence needs is needed in order to restore dignity and hope. A hopeful generation is apt to plan for the future, their health and lives including HIV prevention and treatment. Once again faith organisations have a role to play in ART roll-out. Intervention components that enhance spiritual peace can counteract negative effects of stigma and depression in PLWHA [69,70]. There is a gap the health care workers cannot fill hence the need to integrate faith organisations in the health system.

The findings of the current study need to be considered within several constraints and limitations. A study such as this is limited by virtue of a small sample. Data was collected from nine individuals from three organisations which mean that the findings of this study may not be generalized to other NGOs. An additional and important limitation is that due to the qualitative nature of the study, the quality of the research is heavily dependent upon the prior knowledge and experience of the researcher. Only health care provider perspectives were explored and further research could shed light on patient perspectives.

## Conclusion

In summary, our study revealed that health care workers are confronted by different patient behaviours they fear are undermining ART roll out. Individual or patient behaviour is often thought of as rationale and predictable but the findings revealed that it is heavily influenced by broader socioeconomic, cultural, and environmental factors. New programmes need to address patient behaviour as a complex phenomenon that needs a multi thronged approach encompassing the socio-economic and cultural context of the patient [52]. More validated ART program models are needed that affect social norms and institutions including the integration of traditional medicine and faith based organisations hence our results draw attention to the need for multi-sectoral collaboration between the Department of Health and other departments such as trade and commerce, social welfare, agriculture and public works to ensure a concerted, comprehensive and sustainable ART programme [12,19,69].

## Methods

### Study setting

Three major non-governmental organizations led ART roll-out programmes in KwaZulu-Natal from an early stage: the Southern African Catholic Bishops Conference (SACBC), Hlabisa HIV Treatment and Care Programme (a partnership between the Africa Centre for Health and Population Studies and the Department of Health) and Amangwe village. The SACBC AIDS programme was created as part of the Catholic Church’s response to HIV and AIDS in KZN, South Africa. It was the front runner in ensuring universal access to ART, before the South African Government had made a pledge to ensure universal access to ART [71]. The Africa Centre for Health and Population Studies was established in 1998 with the objective of conducting population research in an ethical manner and to enhance local research capacity. The centre responded in 2004 to the high HIV prevalence by engaging in a partnership with the Department of Health in the delivery of safe, effective, efficient, equitable and sustainable ART to all who needed it in the Hlabisa sub-district, KZN. Staff from both the Department of Health and the Africa Centre worked together to provide HIV treatment and care services in one district hospital and 17 primary health care clinics [72]. Amangwe village was established as a response to the HIV epidemic by the industrial and business community of Richards Bay. The Zululand Chamber of Business Foundation Health and Welfare portfolio recognized the devastating effects of HIV and, after research and consultation with stakeholders, established Amangwe village, an HIV and AIDS intervention that addresses a broad spectrum of HIV-related problems. These are, Ethembeni Care Centre for inpatient and outpatient care of HIV infected patients, an orphan and vulnerable children programme and an outreach, education and training programme. The Centre works alongside the Department of Health, receiving referrals for HIV care and treatment as well as a subsidy [73].

#### Study participants

The participants consisted of programme leaders, doctors and professional nurses working in the three above-named NGOs. These groups were selected as they are the cadres that dealt directly with ART patients from assessment, initiation, monitoring, and management of complications to adherence support. A sample, representative of these sub-groups, was selected by targeting specific sectors [74]. A purposive sample of nine participants was included in the form of three programme leaders who directed and supervised the ART programme, three doctors who were responsible for prescribing, monitoring and dealing with ART complications and three professional nurses who were responsible for monitoring, referring and providing nursing care to patients on ART from the three NGOs. The sample size depended on the saturation of data.

Ethical approval for the study was granted by the UNISA research ethics committee of the faculty of health sciences. Written consent was obtained from directors of the three NGOs involved (Ethembeni Care Centre, Africa Centre and SACBC) and individual audio taped informed consent was obtained from all study participants prior to data collection. Participants were also assured that they could withdraw from the study at any time if they so wished, without penalty.

#### Data collection

A qualitative approach was utilised in an attempt to understand the challenges experienced by NGOs from the subjective perspective of project coordinators, doctors and nurses involved in the roll-out of ART. The complexities, richness and diversity of their work can only be captured by describing what really goes on in their day-to-day work, incorporating the context in which they operate as well as their frame of reference [75]. Data was collected by means of individual face-to-face interviews with participants. The interviews took place in eThekwini, uThungulu and uMkhanyakude districts of KZN in offices on the premises of the relevant NGOs or at clinics between 1 February 2011 and 10 April 2011. None of the professional nurses interviewed had a NIMART qualification nor was initiating ART at the time of interviews despite Department of Health task shifting recommendations having been passed in 2010.

The interview schedule was developed according to Reysoo and Heldens (2007) [76], steps for guide development that include selecting the topic, defining all the aspects of the topic, formulating initial (open ended) questions, determining the kind of questions, determining the logical order of the topics/questions, preparing the introduction and the end and preparing the interview technical indications. Individual face-to-face interviews were held in English in the afternoon between 14.00 and 16.00 when the clinics would be less busy. A semi-structured format was followed and each interview lasted about 20 minutes [77]. The central question was the same, with probes and clarifications, while recording was uniform [74]. The interviews were recorded on an audio recorder with the permission of the interviewees. The central question was “*What are the challenges you experience with regards to patient behaviour in the roll-out of* ART*?”* This question was followed by probing questions based on the initial response from the participant. Notes were written down on the interview schedule during the interview and written up during the transcription to capture the researcher’s own observations. The observations made during data collection assisted the researcher during data analysis in providing additional insight into emergent themes and sub-themes.

Each taped interview was typed by the researcher in the form of a verbatim transcript. The researcher typed the transcripts within two days of the interview and completed the transcripts of one NGO before interviews with the next one.

#### Coding and analysis

Transcribed interviews were entered into ATLAS/ti [78].The transcribed data was independently coded by two investigators who were trained in qualitative data methods. Content analysis was done to explore in detail for common themes and these were then established into units of meaning or codes [75]. Following the traditions of qualitative analyses, the investigators read the transcripts multiple times to identify major themes and then discussed identified themes and came to consensus on coding.

Member checking was also done by having research participants review, validate and verify researcher’s interpretations and conclusion [79]. In addition, triangulation which denotes the use of more than one data source that is programme coordinators, doctors and nurses was also utilised [80].

## Competing interests

The authors declare that they have no competing interests.

## Authors’ contributions

JM, CM, RE, RL and MLN contributed to conception and design, drafting and critical revision of the article. JM, CM and RL undertook acquisition, analysis and interpretation of data. All authors read and approved the final manuscript.
